# The macrogol revolution in the treatment of chronic constipation. A short history of laxatives

**DOI:** 10.3389/fphar.2025.1662224

**Published:** 2025-09-01

**Authors:** Enrico Stefano Corazziari

**Affiliations:** Department of Gastroenterology, IRCCS Humanitas Research Hospital, Rozzano, Italy

**Keywords:** macrogol, polyethylene glycol, constipation, laxatives, childhood, elderly, pregnancy

## Abstract

The difficulty of treating constipation has accompanied humanity through the centuries. Until 1990, numerous remedies were proposed to alleviate the condition; however, due to their aggressive nature or side effects, they were mainly used occasionally or for short periods. When used chronically, there was a high risk of adverse events, sometimes even severe ones. Macrogol, a polyethylene glycol polymer of 3,500–4,000 Daltons, due to its unique physicochemical water binding properties, has revolutionized the treatment of constipation. Macrogol bound to water molecules passes through the entire gastrointestinal tract without being absorbed and metabolized, and without causing significant absorption of water or electrolytes. It is not toxic and does not affect the colonic mucosa. Ultimately, the macrogol-water structure remains unchanged during its transit through the gastrointestinal tract, and, carrying its bound water, increases the luminal volume in the colon with a scarce osmotic effect in the gut lumen. Notably, it has mild adverse effects and no severe adverse effects even when administered long-term or in large amounts. Macrogol changed the paradigm of constipation treatment in the ‘90s. In the macrogol era, it is now possible to initiate treatment in functional, organic, and secondary constipation. In the macrogol era, the chronic constipation algorithm has shifted to become therapeutic first, then diagnostic, without risks for the patient, who can immediately benefit from the treatment. Macrogol offers the possibility to perform long-term treatment, to be safely used in children, in elderly subjects, during pregnancy, and in the presence of irreversible secondary and organic constipation.

## The pre-macrogol era

Constipation is considered a symptomatic manifestation that may present as a reduced frequency of bowel movements, but also as difficulty in defecation or a sensation of incomplete rectal evacuation, regardless of bowel movement frequency. For centuries, however, physicians and lay people regarded constipation as a symptom identifiable mainly by a reduced frequency of bowel movements.

The millennia-long history of constipation therapy, reported since the Egyptian papyrus (Ebbell, 1937) to the 20th century medical literature, was characterized by the use of various means and substances.

These included evacuative enemas up to the end of the 19th century, castor oil (ricinoleic acid), calomel, strychnine, silver nitrates, ipecac, aloe, black mustard seed, rhubarb root, and hyoscyamine (Proceedings of the Chicago Medical Society, 1863). Since then, laxative herbs (mainly containing anthraquinones), non-absorbable oils, and phenylmethane derivatives have become more popular.

All these treatments aimed to induce bowel evacuation but failed to consider the numerous and diverse pathophysiological mechanisms—which were largely unknown at the time—except in cases of constipation secondary to mechanical obstructive or neurological causes, or due to psychiatric and behavioral patterns of fecal retention. In the 19th century, it became evident that the most prevalent condition of constipation was non-organic and could last for years. The available treatments, while inducing evacuation, did not provide satisfactory therapeutic outcomes and often caused undesirable side effects (Tramonte et al., 1997).

It was then that “habitual” constipation, with a chronic duration, began to be recognized as a distinct clinical entity, with its most important pathophysiological characteristics gradually being outlined. Clinical observation associated the lack of dietary fibers and a constantly purged and empty colon with constipation, indicating that the colon needs to be distended by luminal contents to react with peristaltic contractions and distal fecal displacement. Evidence that bran is helpful in constipation because of its fiber content will be given in 1934–1935 (Olmsted and Curtis, 1934; Olmsted and Curtis, 1935).

In the 19th century, the belief emerged that many disorders and diseases were caused by the increasing prevalence of constipation, which was attributed to dietary changes towards more refined foods, reduced physical activity, and the fast-paced lifestyles associated with urbanization.

Health manuals of the time emphasized that the best way to remain healthy was to have a daily bowel movement (Root, 1856).

The discovery that infections were caused by germs, that these germs led to the putrefaction of intestinal contents, and that the metabolism of protein residues generated highly toxic substances capable of being absorbed, led, by the end of the 19th century, to the development of the theory of autointoxication (Bouchard, 1906) resuming the putrefaction theory of the Egyptians (Ebbell, 1937).

This theory was widely used to explain various disorders and diseases that showed no demonstrable organic alteration, and constipation associated with autointoxication came to be considered, by many physicians and much of the population, the most insidious disease: *the disease of diseases* (Whorton, 2000).

In the first 3 decades of the 20th century, in order to ensure daily bowel movements and prevent constipation, doctors recommended diets rich in fruits, vegetables, and whole grains, increased physical activity, and advised against suppressing the urge to defecate (Burkitt and Trowell, 1960).

At the beginning of 20th century, bran-based whole grain cereals and yogurts were introduced to the market to facilitate evacuation and prevent autointoxication.

Laxatives, mainly based on anthraquinones or phenolphthalein, also became highly popular, and various remedies for rectal stimulation or colonic irrigation were marketed, as well as machines for abdominal massage.

The concern about constipation was so great that very many patients accepted colectomy as a solution to eliminate autointoxication, as proposed by the London surgeon Lane (Lane, 1913).

In the early years of the 20th century, two important mechanisms of constipation were identified: slow colonic transit and the difficulty in expelling stool from the rectum, named “*dyschezia*” (Hurst, 1908; Hurst, 1909).

For patients with dyschezia, the preferred therapeutic approach focused on rectal stimulation through enemas or laxative suppositories. For those with slow colonic transit, even as late as 1968, *the best type of laxatives had not been established* (Hinton and Lennard-Jones, 1968).

In the second half of the 20th century, the chronic use of laxatives was widely demonized due to a series of misconceptions and as a reaction to the improper and excessive use of these drugs during the first half of the century. Many of these misconceptions stemmed from incorrect usage, largely based on self-medication, which can lead to side effects, some of them severe.

*Melanosis coli*, a condition resulting from prolonged use of anthraquinone-based laxatives, was long considered a sign of damage but has since been shown to have no pathological significance (Badiali et al., 1985). The so-called “cathartic colon,” which was mistakenly thought to be an irreversible and serious condition involving degeneration of the intramural nerve plexuses and the colonic musculature due to prolonged laxative use, has been now dismissed as a pathological entity (Müller-Lissner, 1996).

In 1968, lactulose became available as a laxative that was both effective and reliable due to its safety (Wesselius-de Casparis et al., 1968). However, its use for chronic constipation remained limited because of the undesirable side effects of bloating and flatulence, and it was recommended mainly for hepatic encephalopathy, during pregnancy, and in childhood.

Until 1990, the treatment of constipation aimed to achieve bowel evacuation either by stimulating motor activity through colon distension with bulk-forming agents, or by using stimulant laxatives, or osmotic laxatives based on salts or disaccharides (Roth and Beschke, 1988).

For several years, it had already been known that the intestine could be mechanically emptied by orally administering large volumes—up to 10 L—of saline solution (Smith et al., 1978). However, it was only in 1980 that it was demonstrated that a complete bowel washout could be achieved with 3–4 L of a polyethylene glycol solution (renamed macrogol for gastroenterological use), without disrupting the body’s balance and without relevant side effects (Dav et al., 1980).

## Polyethylene glycol and macrogol, a water-binding agent

### Physico-chemical properties

**FIGURE 1:**
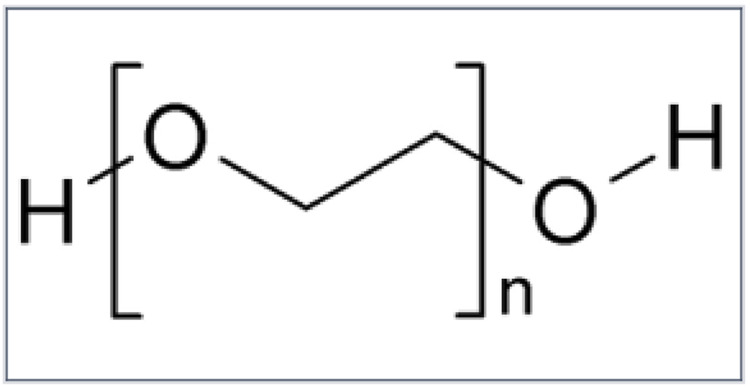
The linear copolymer of ethylene oxide (CH_2_CH_2_O) and water (H_2_O).

Polyethylene glycol is a linear copolymer of ethylene oxide (CH_2_CH_2_O) and water (H_2_O). Depending on the degree of polymerisation, PEG is liquid up to a molecular mass of 400 Da and solid above a molecular mass of 1,000 Da (Figure 1).

**FIGURE 2:**
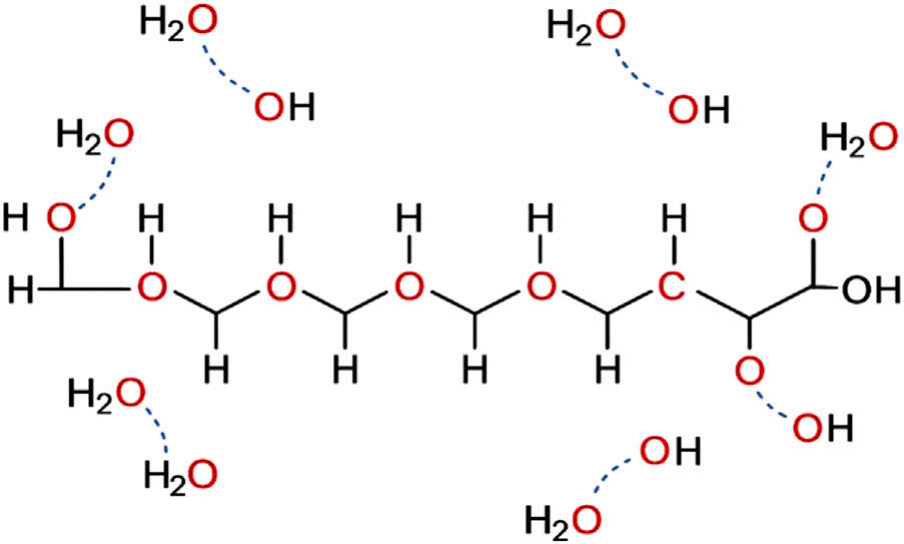
Interaction of macrogol 3,500–4,000 with water molecules.

Macrogol, a polymer of polyethylene glycol of 3,500–4,000 Da, is an inert, electrically neutral substance, non-metabolized and non-fermentable by intestinal microbiota. Macrogol binds to the hydrogen atoms of water molecules through its polar oxygen atoms (Figure 2).

The characteristic properties of macrogol with water give rise to a new macrogol-water structure that passes through the entire gastrointestinal tract without being absorbed and without causing significant absorption of water or electrolytes (Davis et al., 1980). Even when macrogol is administered at high volume for large bowel cleansing, there are no volume overload or depletion, serum electrolyte imbalance, toxicity, or alterations of colonic mucosal histology (Michael et al., 1985). Ultimately, the macrogol-water structure formed during the preparation of the solution in the glass, after ingestion, remains unchanged during its transit through the gastrointestinal tract, and, carrying its bound water, increases the luminal volume in the colon with a scarce osmotic effect in the gut lumen.

PEG does not interfere with colonic fluid absorption or with the ability of the colonic mucosa to generate and sustain steep electrochemical gradients. As a result, increased fecal fluid produced by ingestion of 53 g/d of PEG contained only 4 and 6 mEq/d of sodium and potassium, respectively, indicating that laxative doses of PEG would not be likely to cause electrolyte depletion (Hammer et al., 1989).

Sources of the water content in the stool are 80% from microbes and 10% (dilution water) from mucus,soluble components, and bound to electrolytes. Macrogol hydrates and decreases stool consistency through increments of dilution water (Chaussade, 1999).

Diarrheal stools produced by PEG had lower levels of liquidity than diarrheal stools produced by other osmotic laxatives, such as lactulose, milk of magnesia, sodium sulfate, and phenolphthalein (Schiller et al., 1988).

Pharmacokinetic investigations have shown an absorption rate of less than 1.6% for macrogol (Muller-Lissner, 1999).

As a result, macrogol exerts its ultimate action in the large bowel by increasing the fecal volume and decreasing stool consistency. Consequently by distending the walls, promotes the propulsive contractions (Corsetti et al., 2021) and accelerates transit, mainly in the left part of the colon and the rectum (Corazziari et al., 1996); by counteracting the dehydrating action of the large bowel, avoids retention of dry pellety or hard stools in the rectum from where they are dealt with difficult and tiring straining effort. In addition, avoiding rectal fecal accumulation prevents fecal stasis in the large bowel and interrupts the vicious cycle of subsequent fecal dehydration.

The fact that macrogol had been known for over a century as a non-toxic substance—widely used as an excipient and characterized by being inert, non-fermentable by gut microbiota, and non-absorbable by the gastrointestinal tract, while retaining water within the lumen—alongside strong evidence of its high efficacy and safety when used for bowel cleansing before colonoscopy, led to its use in low volume daily doses for the treatment of chronic constipation.

## The macrogol era

In 1991, a study conducted on a limited number of subjects for no more than 1 week reported the favorable effect of macrogol in constipated patients (Baldonedo et al., 1991).

In 1996, the first Randomized Controlled Trial (RCT) demonstrated the therapeutic efficacy of low daily doses of macrogol in the treatment of chronic constipation over a period of 8 weeks (Corazziari et al., 1996).

In 2000 a second RCT confirmed the therapeutic efficacy and demonstrated the safety of low daily doses of macrogol over 6 months (Corazziari et al., 2000), and since then, other RCTs confirmed these results both in short (DiPalma et al., 2000; Cleveland et al., 2001) and long-term treatment (Dipalma et al., 2007).

In those same years, constipation, previously considered a secondary symptom attributed to various presumed factors such as fiber deficiency, poor bowel management, or a psychosomatic issue, came to be recognized as a primary and chronic functional disorder requiring ongoing treatment (Drossman et al., 1994). “However, in 2000 and again in 2002, meta-analyses and reviews in the gastroenterology literature either did not acknowledge the use of macrogol for the treatment of chronic constipation or did so with caution (Locke et al., 2000; Jones et al., 2002). It was not until 2005 that its indication was definitively taken seriously into consideration (Ramkumar and Rao, 2005).

An important contribution to the proper management of chronic constipation came from a literature review, which highlighted that laxatives, when used appropriately, do not have negative consequences; that water intake and physical activity do not have substantial definitive therapeutic effects; and that fiber is not a cure-all for the prevention or treatment of constipation (Müller-Lissner et al., 2005).

The availability of macrogol, a laxative with no relevant and no serious adverse effects, has significantly changed the approach to and the management of patients with chronic constipation (Corazziari ES La stipsi cronica, 2012).

In the pre-macrogol era, the diagnosis of chronic constipation first required the exclusion of organic causes, typically through diagnostic investigations (radiological before 1980 or endoscopy), often performed after the use of cathartic laxatives or enemas, which carried the risk of side effects and potential complications. This was particularly concerning in patients with colonic diverticula, substenotic luminal conditions, or in those undergoing repeated evacuative enemas.

The diagnostic pathway was therefore complex, not without risks, and carried the possibility of iatrogenic harm. Precisely because of this complexity, it was usually applied only after careful evaluation, following an algorithm that involved an initial diagnostic phase followed by a therapeutic phase.

In the macrogol era, except in cases of intestinal perforation or complete bowel obstruction, it is now possible to initiate treatment with macrogol both in chronic constipation secondary to organic causes and in functional constipation. This allows the patient to undergo diagnostic investigations with the bowel free of fecal material and without time constraints.

In the macrogol era, the chronic constipation algorithm has shifted to become therapeutic first, then diagnostic, without risks for the patient, who can immediately benefit from the treatment (Corazziari ES La stipsi cronica, 2012).

Macrogol prescription should be tailored to the patient’s clinical condition of constipation and habits to manage it. Upfront finding of fecal rectocolonic loading or fecaloma is an indication for high volume macrogol to free the fecal overload and subsequently start a daily low volume treatment and decide whether to perform a differential diagnostic investigation or to maintain a daily low volume macrogol treatment. In the absence of fecal rectocolonic loading, macrogol treatment can be started with a daily low-volume prescription, and subsequently, there is time to decide whether to perform a differential diagnostic investigation.

## Preferential use of macrogol in different conditions

For ease of use and its physicochemical properties, macrogol has been recommended as a preferred treatment for chronic constipation in various conditions.

For example, in patients undergoing treatment with opioid analgesics (De Giorgio et al., 2021) or other medications that cause constipation.

Constipation during pregnancy can be treated with fiber, lactulose or macrogol. Bloating, diarrhea and loose stools are less frequently reported with macrogol than with lactulose (Kothari et al., 2024; Raj et al., 2024).

When considering the use of laxative medications in the elderly, the choice of the preparation and dosage is critical, as a cathartic effect with the production of liquid stools is associated with the risk of fecal incontinence due to the high prevalence of sensory and motor anorectal-pelvic dysfunctions in this age group. Therefore, stimulant contact laxatives and saline osmotic laxatives should be avoided as first-line options, since their evacuative response is unpredictable and may produce a cathartic effect even at low doses.

For elderly patients, macrogol is highly effective, with no risk of severe side effects and without interfering with the absorption or metabolism of other medications. Macrogol is particularly useful for hydrating the stool and facilitating defecation, which is often difficult for elderly individuals (Whitehead et al., 1989) and requires significant effort due to the presence of dry and hard stools. Additionally, by adjusting the daily dose of macrogol, it is possible to soften stool consistency without causing diarrhea. Furthermore, the product’s safety profile allows its use even when an organic pathology is suspected and during the diagnostic process, before a definitive diagnosis has been established.

Macrogol is also preferred over other laxatives, for example, in patients with colonic diverticula or with inflammatory colorectal diseases in whom a stimulant effect on colonic contractility or the gaseous distension of disaccharides should be avoided.

Constipation in patients with neurological diseases is generally chronic and irreversible, with a risk of fecaloma formation, bowel obstruction episodes, colonic distension, and perforation. The primary goal is to prevent the formation of fecalomas and to maintain a normal defecation pattern. In patients with impaired continence, the use of stimulant laxatives and saline osmotics should be avoided due to the unpredictability of their cathartic effect. Moreover, if misused, they can cause electrolyte imbalances, asthenia, and muscle cramps (Corazziari et al., 1987). Bulk-forming agents are contraindicated in cases of luminal strictures and are less effective in constipation caused by delayed rectal transit or disordered defecation (Badiali et al., 1995).

The therapeutic effectiveness of macrogol in the treatment of constipation has been reported in diabetic patients with autonomic neuropathy (Rossol, 2007), in patients with neurological disorders, Parkinson’s disease, and multiple system atrophy (Eichorn and Oertel, 2001). Even in patients with disordered defecation, if the stool is hard and dehydrated, it is advisable to administer macrogol solutions, which normalize stool consistency and make it easier for the anorectal system to manage.

## Prescription for adults

The prescription is an important part of macrogol therapy because one dose does not fit all! Macrogol should be taken daily at the minimum effective dose that produces well hydrated stools that are passed without effort. The method of administration—whether in a single dose or divided into multiple doses throughout the day—has not been established by specific studies. However, based on clinical experience, I believe that, in order to maintain treatment compliance, macrogol can be taken at the time and in the manner preferred by the patient.

The daily dose cannot be predetermined but must be identified by the patient within a few days by either increasing or decreasing it. In clinical practice, it is good practice to instruct the patient to increase the daily dose of macrogol if hard stools persist, and conversely, to decrease it in the presence of unformed stools. Therefore, the dosage—by adjusting the volume of solution taken daily either up or down—should be individually tailored to each patient’s response.

On average, most patients with chronic constipation benefit from 10 to 30 g of macrogol per day, but no adverse events have been reported by those who need greater daily doses. In some cases, taking macrogol every other day is sufficient.

## Macrogol in childhood

Constipation in childhood, except for the rare cases of Hirschsprung’s disease and cases secondary to typically neurological causes, is functional. Unlike adult constipation, it is characterized by stool retention behavior, with feces accumulating in the rectum and progressively in the colon, leading to the development of megarectum and megacolon.

In a child presenting with fecal impaction, the first and essential step is to relieve the rectum and colon from the obstruction and distension caused by the retained stool. In the pre-macrogol era, disimpaction of the colon and rectum was performed through manual maneuvers or evacuative enemas. However, since the introduction of macrogol, oral therapy has become preferred due to its efficacy, safety, and less invasive nature. Compared to transanal treatments, oral therapy improves adherence because it is better accepted by both the child and the parents, creates less aversion to therapy, and reduces parent-child conflict in the management of bowel movements.

Bowel clearance can be achieved by administering macrogol at a dose of 1–1.5 g/kg/day for up to 6 consecutive days (Youssef et al., 2002; Candy et al., 2006). After satisfactory colonic emptying and softening of the stool are achieved—possibly by repeating the initial treatment if necessary—maintenance therapy with oral macrogol should be initiated to keep stools soft and evacuations painless.

The dose of macrogol for long-term maintenance therapy ranges from 0.4 to 0.8 g/kg/day and must always be tailored to the individual case, adjusting it according to the response in terms of stool consistency and evacuation frequency (Candy and Belsey, 2009; Nurko et al., 2008).

## Conclusions

For centuries, the treatment of constipation has focused on symptomatic relief, relying on harsh, inconsistent remedies not devoid of adverse effects. Macrogol changed the paradigm of constipation treatment in the ‘90s. Since then, it is possible to initiate treatment with macrogol both in functional constipation and in chronic constipation secondary to organic causes. This allows the patient to undergo diagnostic investigations with the bowel free of fecal material and without time constraints. In the macrogol era, the chronic constipation algorithm has shifted to become therapeutic first, then diagnostic, without risks for the patient, who can immediately benefit from the treatment. Macrogol offers the possibility to perform long-term treatment, to be safely used in children, in elderly subjects, during pregnancy, and in the presence of irreversible secondary and organic constipation.

## Data Availability

The original contributions presented in the study are included in the article/supplementary material, further inquiries can be directed to the corresponding author.
